# Glucose restriction in *Saccharomyces cerevisiae* modulates the phosphorylation pattern of the 20S proteasome and increases its activity

**DOI:** 10.1038/s41598-023-46614-x

**Published:** 2023-11-08

**Authors:** Renata Naporano Bicev, Maximilia Frazão de Souza Degenhardt, Cristiano Luis Pinto de Oliveira, Emerson Rodrigo da Silva, Jéril Degrouard, Guillaume Tresset, Graziella Eliza Ronsein, Marilene Demasi, Fernanda Marques da Cunha

**Affiliations:** 1 Departamento de Bioquímica, Escola Paulista de Medicina, Universidade Federal de São Paulo, São Paulo, SP Brasil; 2 Departamento de Física Experimental, Instituto de Física, Universidade de São Paulo, São Paulo, SP Brasil; 3 Departamento de Biofísica, Escola Paulista de Medicina, Universidade Federal de São Paulo, São Paulo, SP Brasil; 4 Université Paris-Saclay, CNRS, Laboratoire de Physique des Solides, 91405 Orsay, France; 5 Departamento de Bioquímica, Instituto de Química, Universidade de São Paulo, São Paulo, SP Brasil; 6 Laboratório de Bioquímica, Instituto Butantan, São Paulo, SP Brasil

**Keywords:** Proteasome, Saccharomyces cerevisiae

## Abstract

Caloric restriction is known to extend the lifespan and/or improve diverse physiological parameters in a vast array of organisms. In the yeast *Saccharomyces cerevisiae*, caloric restriction is performed by reducing the glucose concentration in the culture medium, a condition previously associated with increased chronological lifespan and 20S proteasome activity in cell extracts, which was not due to increased proteasome amounts in restricted cells. Herein, we sought to investigate the mechanisms through which glucose restriction improved proteasome activity and whether these activity changes were associated with modifications in the particle conformation. We show that glucose restriction increases the ability of 20S proteasomes, isolated from *Saccharomyces cerevisiae* cells, to degrade model substrates and whole proteins. In addition, threonine 55 and/or serine 56 of the α5-subunit, were/was consistently found to be phosphorylated in proteasomes isolated from glucose restricted cells, which may be involved in the increased proteolysis capacity of proteasomes from restricted cells. We were not able to observe changes in the gate opening nor in the spatial conformation in 20S proteasome particles isolated from glucose restricted cells, suggesting that the changes in activity were not accompanied by large conformational alterations in the 20S proteasome but involved allosteric activation of proteasome catalytic site.

## Introduction

The catalytic unit of the proteasome named 20S is the proteolytic core of the ubiquitin proteasome system (UPS), the primary pathway through which cells control the levels of most intracellular proteins, in a regulated manner. UPS activity is crucial to proteostasis^1^. Indeed, UPS dysfunction was reported to be involved in the pathogenesis of human diseases, especially those characterized by protein misfolding and aggregation such as Parkinson's and Alzheimer's disease^1^. Conversely, genetic or pharmacologic interventions that improve the UPS activity either in cellular models or in diverse organisms were reported to increase lifespan and longevity, respectively^2–6^.

The 20S proteasome complex is a 700 kDa hollow cylinder, composed of 28 subunits arranged in four heptameric rings: two central β rings flanked by two rings consisting of α-type subunits^7^. The catalytic sites are located into the β rings, while the α rings regulate the opening of the catalytic chamber gate upon interaction with the regulatory units^7^. The 20S proteasome (PT 20S) has a broad substrate specificity, hydrolyzing peptide bonds after basic (trypsin-like activity), hydrophobic (chymotrypsin-like activity) and acid (caspase-like activity) amino acids^8^.

The most studied mechanism through which proteasome activity is modulated is the coupling to regulatory units^9^. However, the free 20S proteasome is present in considerable amounts in cells (more than 50% of the total proteasome particles is in the free form in live mammalian cells in culture;^10^). In addition it has been shown that the free 20S proteasome is capable of degrading specific groups of proteins, such as intrinsically disordered proteins^11,12^, oxidized proteins^13^ or even ubiquitinated proteins^14^. Of note, an array of post-translational modification sites was identified in the 20S proteasome subunits, many of which not yet characterized as to its impact on proteasome activity^9,15^. Phosphorylation and S-glutathionylation are examples of post-translational modifications that control proteasomal activity^16,17^.

The proteostasis system in general and proteasome activity in particular were shown to be positively impacted by caloric restriction (CR)^18–22^. Caloric restriction is a nutritional intervention of reduction of calories ingestion without malnutrition^23^. A robust collection of experimental data in animal models and humans shows that CR is capable of promoting health benefits, sometimes accompanied by longevity extension in some organisms^24–28^. In the yeast* S. cerevisiae*, glucose reduction in the culture medium is used as a calorie restriction model^28^. Experimental data has shown that growing *S. cerevisiae* under glucose restriction (GR) extends its replicative and chronological life spans and decreases the accumulation of oxidatively damaged proteins^18,29^. Those effects are generally accompanied by several metabolic changes, including a shift from fermentation to respiration^28,30^, enhanced resistance to stress^31^, and increased clearance of damaged macromolecules by means of autophagy^32^. In a previous work, we demonstrated that the positive impact of glucose restriction on proteasome activity is conserved in the yeast *S. cerevisiae,* as extracts of cells grown under glucose restriction had increased 20S proteasome activity, which was not due to increased proteasome amounts in the cell extracts^18^.

In the present work we went further to investigate the effects of glucose restriction on the proteasome 20S and discovered that glucose restriction-induced increase in proteasome activity is maintained after proteasome isolation from cellular extracts. Interestingly, structural investigation of the 20S proteasome did not reveal glucose restriction-induced changes in the opening of the gate nor in spatial conformation of the particle, suggesting that glucose restriction-induced improvement in proteolytic capacity is not the result of gross structure alterations. We identified two amino acid residues in the α5-subunit (Threonine 55 and/or Serine 56) that are phosphorylated in the glucose restriction proteasomes (PT-GR) but not in the control proteasomes (PT-C). Importantly, in silico modeling indicates that phosphorylation at these sites assists the interaction of α-synuclein with the α ring of the 20S proteasome, which may be involved with the increased proteolysis capacity of proteasomes isolated from cells cultured under glucose restriction.

## Results

### Glucose restriction increases 20S proteasome activity, without affecting its coupling to the regulatory unit 19S

**Figure 1:**
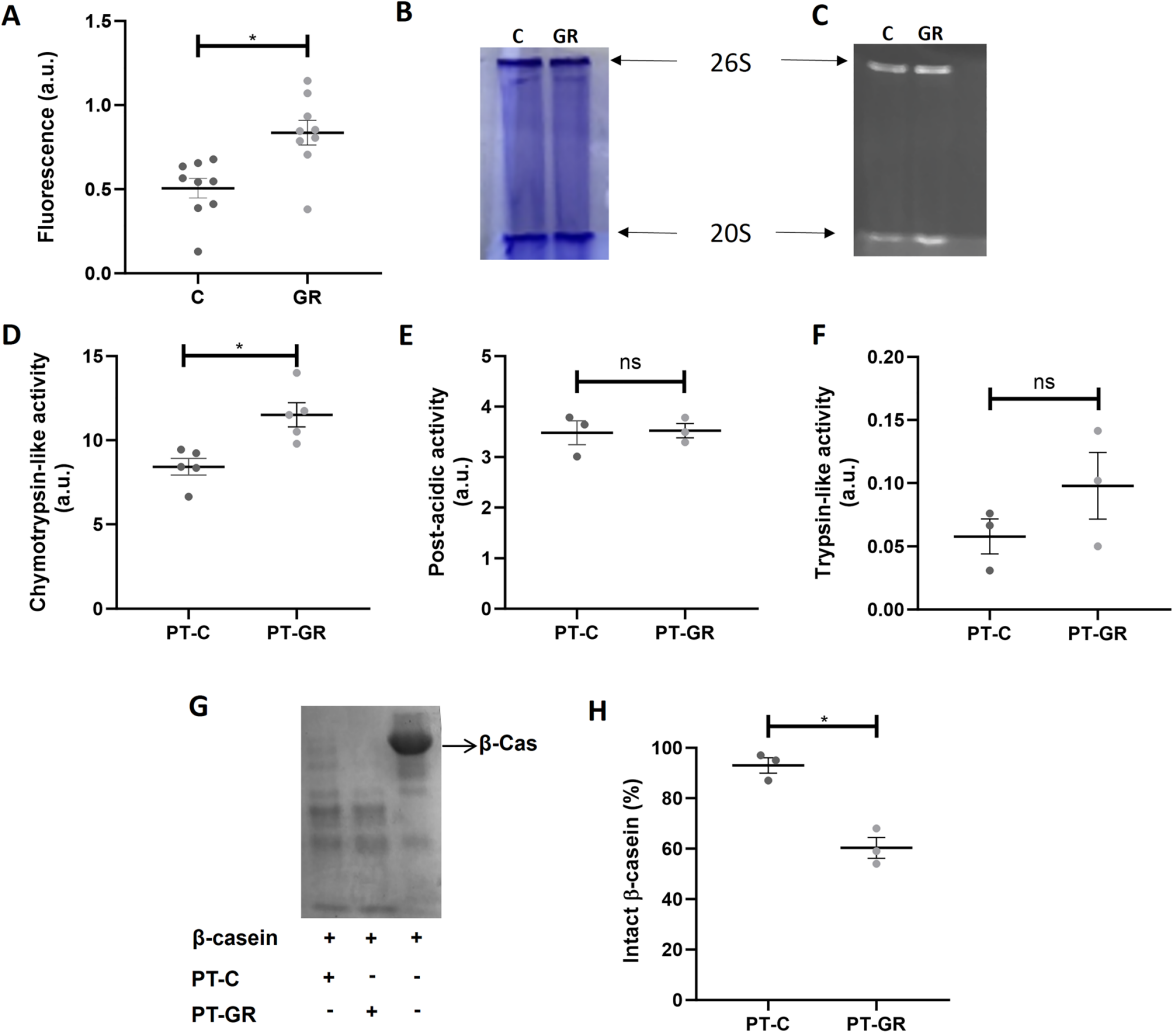
Effect of glucose restriction on proteasome activity. (**A**) Proteasome activity in cellular extracts was assessed by incubating 50 µg of protein from control (**C**) or glucose restriction (GR) samples with 125 mM Suc-LLVY-AMC fluorogenic substrate as described in Methods. Each group represents nine independent assays ± standard deviation. *P < 0.05 significantly different from the control group; unpaired t test. a.u. = arbitrary units. (**B**–**C**) Proteins from cellular extracts obtained from cells grown under C or GR condition were separated by native electrophoresis as described in Methods. The gel was stained with Coomassie (**B**) and in-gel activity was assessed by spreading 100 µM Suc-LLVY-AMC onto the gel (**C**). (**D**–**F**) The peptidase activities of 20S proteasomes purified from C or GR cell extracts was evaluated by incubating 1 µg of isolated proteasomes with a fluorogenic substrate specific for each of the three catalytic sites of the proteasome: (**D**) 125 µM of Suc-LLVY-AMC for chymotrypsin-like activity, (**E**) 200 µM Z-LLE-AMC for post-acidic activity or (F) 200 µM Z-ARR-AMC for trypsin-like activity. Data represent 5 independent experiments for chymotrypsin-like activity and 3 independent experiments for trypsin-like and post-acidic activities ± standard deviation. * P < 0.05 significantly different from the control group; unpaired t test. (**G**) Proteolytic activity of proteasomes isolated from C or GR cells was assessed by incubating 5 µg of proteasomes with β-casein. Samples were submitted to electrophoretic separation in a 15% denaturing gel subsequently stained with Coomassie blue as described in Methods. (H) Quantification of intact β-casein. Each group represents 3 independent experiments ± standard deviation.

We previously showed that cellular extracts from *S. cerevisiae* cells grown under glucose restriction had increased proteasome chymotrypsin-like activity when compared to control^18^. In the present work, we replicated the finding that cellular extracts obtained from *S. cerevisiae* cells cultured under glucose restriction present increased 20S proteasome activity when compared with cellular extracts obtained from cells cultured under control conditions (Fig. 1A,C; Fig. S6). Since one of the ways of proteasomal activation is coupling of the 20S catalytic subunit to the 19S regulatory unit, the effect of glucose restriction on proteasome coupling was investigated by submitting cellular extracts obtained from the two different conditions to native gel electrophoresis. No difference in the amounts of proteasomes 20S or 26S was observed when samples from control or glucose restriction groups were compared (Fig. 1B; Fig. S6). Next, we went on to investigate whether this increased activity is maintained in 20S proteasomes isolated by affinity chromatography. The purification protocol yielded highly pure proteasomes as indicated by sodium dodecyl sulphate polyacrylamide gel electrophoresis (SDS-PAGE; Fig. S1). Interestingly, in spite of the long purification process (2 days), 20S proteasomes purified from cells cultured under glucose restriction maintained increased chymotrypsin-like activity when compared with 20S proteasomes isolated from cells cultured under control conditions (Fig. 1D). Of note, post-acidic- and trypsin-like activities were not affected by glucose restriction (Fig. 1E,F).

Model substrates are important tools to assess the specific proteasome activities, but do not always represent the capacity of proteasomes to degrade whole proteins^33^. To check whether increased site-specific activity translated into increased proteolysis, purified proteasomes were incubated with β-casein, a protein reported to be degraded by the 20S proteasome in an ATP- and ubiquitin-independent manner^34^. Our results indicate that proteasomes purified from glucose restricted cells were more able to degrade β-casein when compared with proteasomes isolated from control cells (Fig. 1G,H, Fig. S5C). We also assayed two other proteins described to be degraded by the 20S particle: α-synuclein^35^ and glutaredoxin 2^36^, and found that while α-synuclein was more degraded by 20S proteasomes purified from cells cultured under glucose restriction (Fig. S5A), the same was not observed for glutaredoxin 2 (Fig. S5B). Despite the fact that α-synuclein and glutaredoxin 2 were single replicates, taken together, the results indicate that glucose restriction increases 20S proteasome activity in a site-specific manner, which translates into increased proteolysis towards some, but not all, 20S proteasome substrates.

### Glucose restriction does not affect 20S proteasome spatial conformation

**Figure 2:**
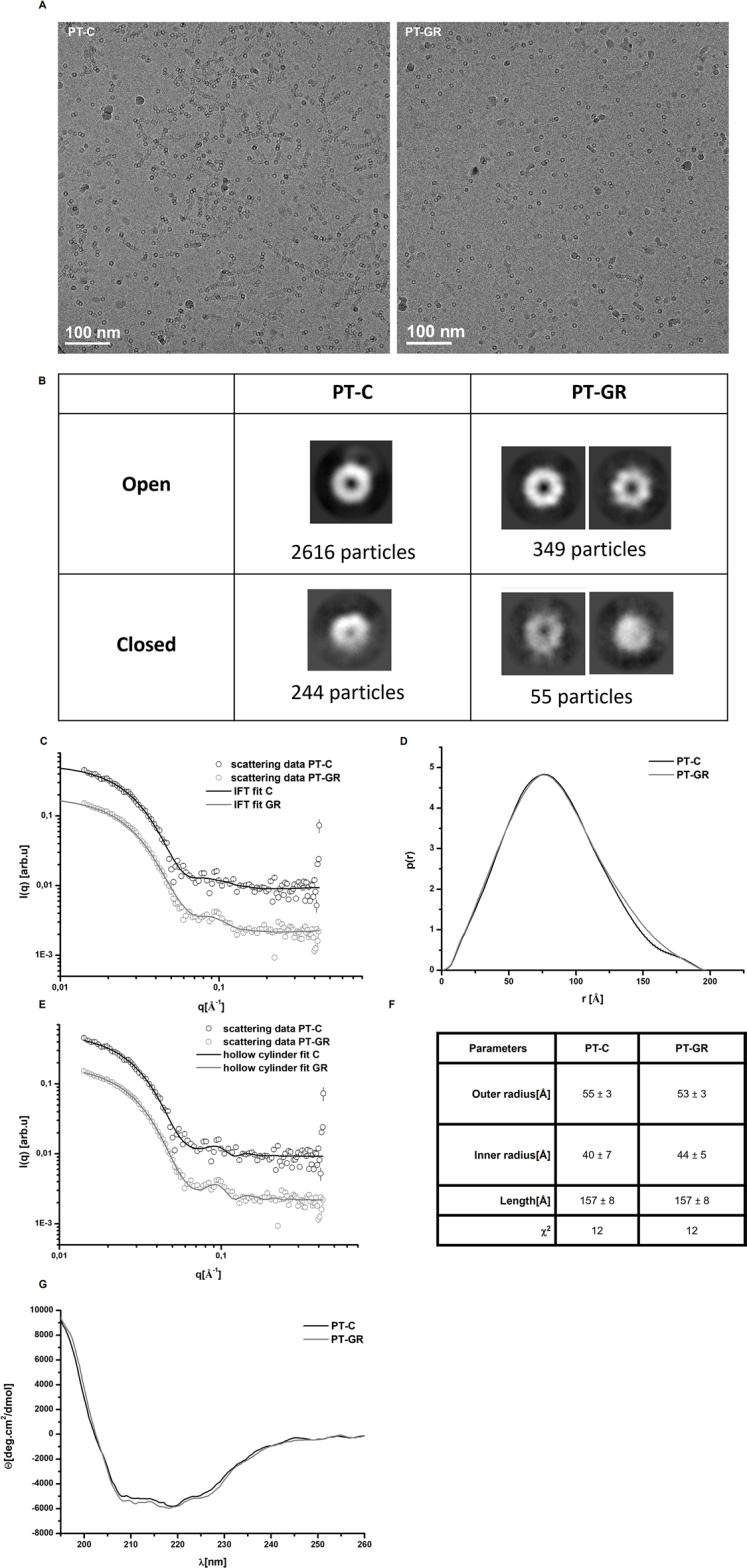
Glucose restriction does not change the conformation of the 20S proteasome. (**A**) Representative cryo-transmission electron microcopy (cryo-TEM) micrographs of 20S proteasome particles isolated from cells cultured under control (PT-C) or glucose restriction (PT-GR) conditions. (**B**) 2D classification of pore opening and frequency distribution in experimental samples performed with cryoSPARC software. (**C**–**F**) PT-C or PT-GR samples were analyzed by SAXS as described in experimental procedures. (**C**) Scattering curves, (**D**) distance pair distribution function, p(r), (E) fitting of scattering curves using a form factor of hollow cylinder, and (**F**) hollow cylinder modelling results. (**G**) Circular dichroism curves obtained for PT-C or PT-GR samples as described in experimental procedures. The data is representative of 2 independent experiments.

Since proteasomes purified from glucose restricted cells have increased activity compared with proteasomes purified from control cells, a set of assays was performed to check whether the differences in activity were accompanied by differences in spatial conformation. The entrance pore of 20S proteasomes was analyzed using images obtained by cryo-electron transmission microscopy (Fig. 2A) after classifying the imaged particles as presenting an open or closed gate (Fig. 2B). The results indicated that 86% of proteasomes in the glucose restricted group presented the gate open (Fig. 2B) while this number was 91% in the control group (Fig. 2B). In addition, the small angle X-ray scattering (SAXS) profile and pair distance distribution function (p(r)) were also determined for glucose restriction or control samples. The p(r) function obtained for both experimental groups is characteristic of short cylindrical particles^37^, and the curves were superposable (Fig. 2C,D), indicating that there is no detectable difference (for the resolution of the SAXS data) in the overall spatial conformation of the 20S particle between control and glucose restriction conditions. To reinforce this result and analyze the dimensions of the proteasome, the intensity curve obtained by the SAXS assay was adjusted using the form factor of a hollow cylinder (Fig. 2E). The adjusted dimensions obtained for PT-C and PT-GR were similar (Fig. 2F), indicating that both samples were composed of cylindrical particles (as reported for the 20S proteasome;^38–40^) of similar dimensions. Finally, the secondary structure analyzed by circular dichroism revealed the superposition of signals from α-helix and β-strands (Fig. 2G) as previously described for the 20S proteasome^41^. The analysis of 20S proteasome particles isolated from control or glucose restricted cells by circular dichroism did not reveal differences between samples, with both experimental groups exhibiting the same profile (Fig. 2G). Together, the results suggest that glucose restriction-induced increase in 20S proteasome activity is not accompanied by significant changes in the particle’s structure.

### Glucose restriction induces phosphorylation of proteasome α5- subunit

Taking into consideration that: i—the effect of glucose restriction on proteasome activity was detected not only in cell extracts but also in purified proteasomes, and ii—no relevant structural change in the 20S proteasome induced by glucose restriction was observed, we decided to investigate the hypothesis that glucose restriction would affect post-translational modifications on 20S proteasome subunits that could, in turn, impact on proteasome activity.

**Figure 3:**
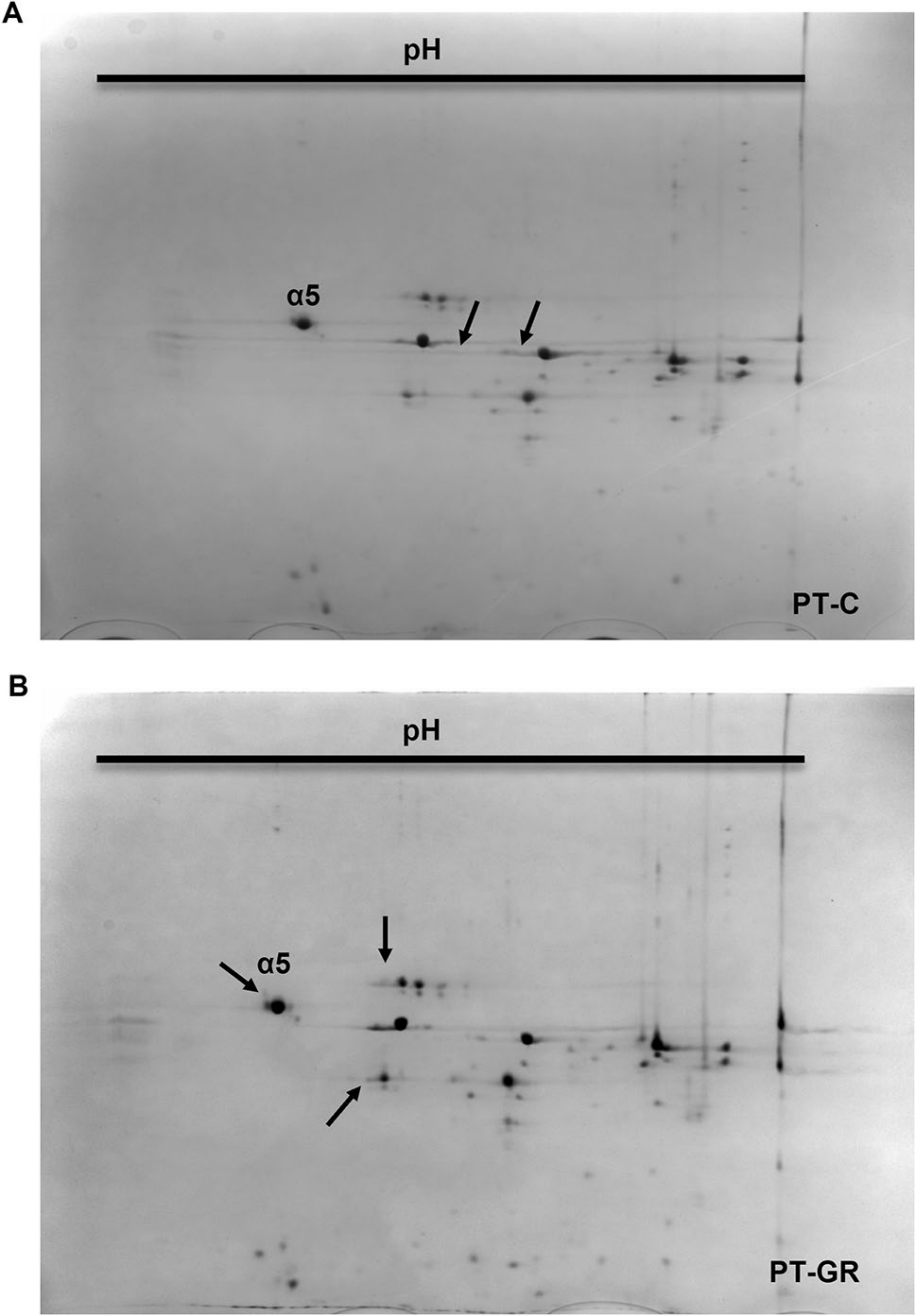
Glucose restriction alters the isoelectric point of some 20S proteasome subunits. One hundred and fifteen µg of proteasomes isolated from (**A**) control (PT-C) or (**B**) glucose restricted (PT-GR) cells were separated according to their isoelectric point with the pH range of 4 to 7 from left to right (first dimension), and size (molecular weight—second dimension). The gels were stained with colloidal Coomassie G-250 and the images were acquired and compared as described in experimental procedures. The arrows highlight differences in proteasome subunits.

**Figure 4:**
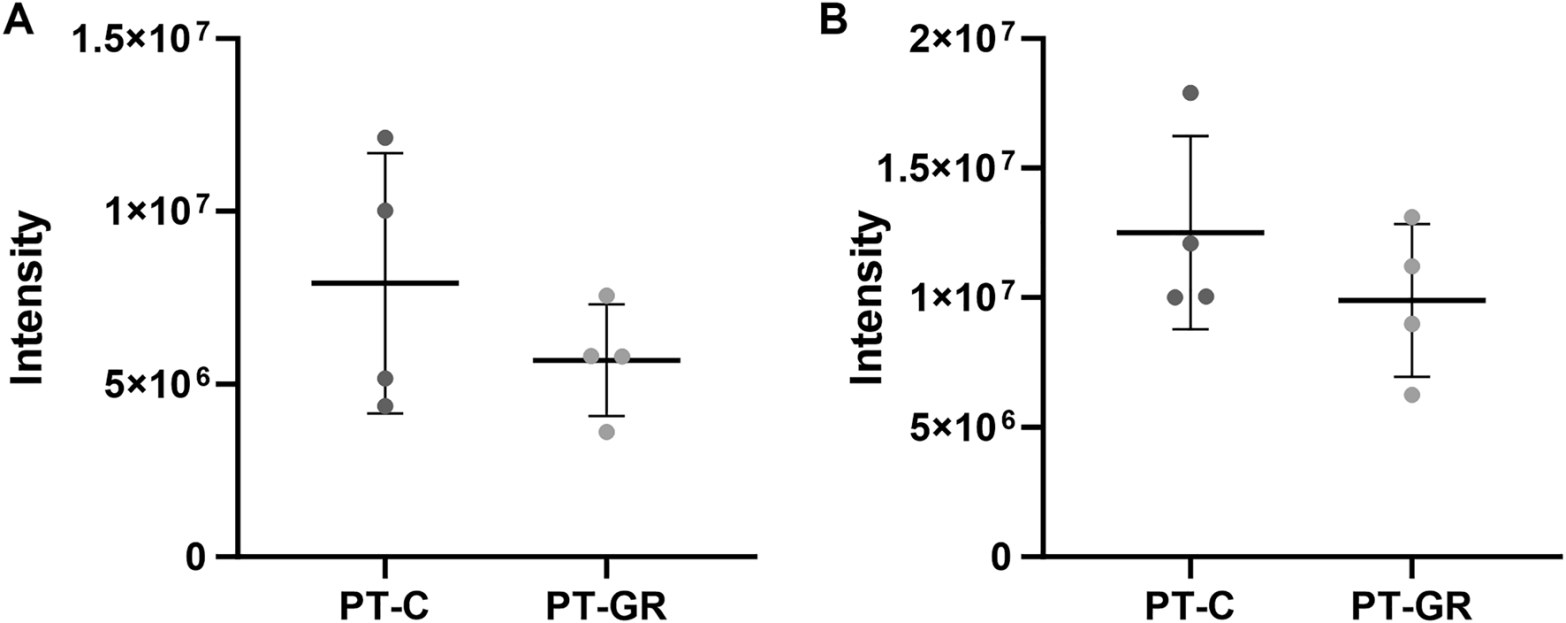
Glucose restriction does not change the amounts of known 20S proteasome interacting proteins. Ten µg of purified proteasomes isolated from control (C) or glucose restricted (GR) cells were digested with trypsin and peptides were analyzed by mass spectrometry as described in experimental procedures. (**A**) Quantitative analysis of Fub1 and (**B**) Blm10 proteins. Protein abundance was quantified by the label-free quantification (LFQ) algorithm, based on the normalized chromatographic peak integrations generated by MaxQuant. Each group represents 4 biological replicates ± standard deviation.

**Figure 5:**
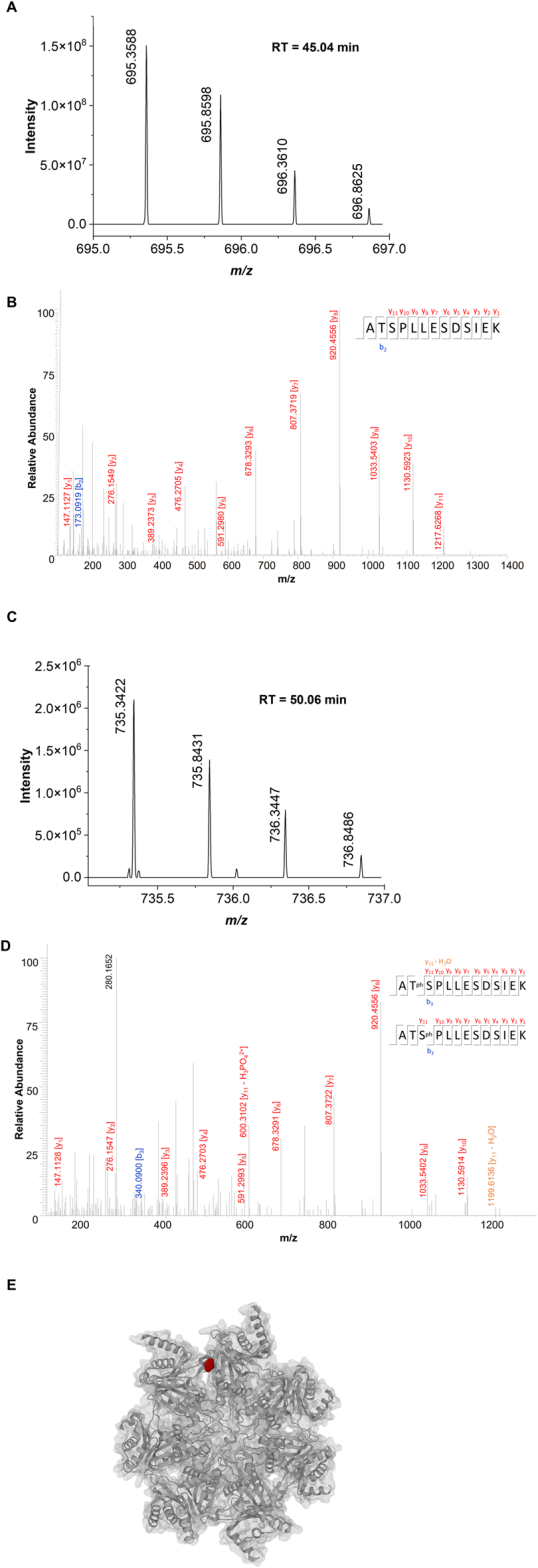
Glucose restriction induces the phosphorylation of at least one residue in α5 proteasome subunit. MS (**A**) and MS/MS (**C**) spectra of the peptide **ATSPLLESDSIEK** without phosphorilation on site 2 or 3. MS (**B**) and MS/MS (**D**) spectra of the peptide **ATSPLLESDSIEK** with phosphorilation on site 2 or 3. (**E**) Top view representation of the three-dimensional structure of the 20S proteasome (pdb entry 3D29). The position of the Thr55 residue is highlighted in red. The image was created on the protein data bank website, combining cartoon and molecular surface visualization^41^.

Purified proteasomes were subjected to two-dimensional gel electrophoretic separation. The assay revealed differences suggestive of post-translational modification between the studied experimental groups (Fig. 3). At least 5 spots detected in the gel presented differences in isoelectric migration patterns suggestive of post-translational modifications when glucose restriction sample was compared with control sample (Fig. 3 and Fig. S2). We went on to perform a more careful analysis of post-translational modifications by directly injecting tryptic peptides from purified proteasomes, without prior electrophoresis separation, into the mass spectrometer. The obtained results further confirmed that our purification protocol yielded pure samples, since only 47 non-proteasomal proteins were detected and of those, only Fub1p and Blm10p were consistently detected in most of the biological replicates (Table S1). No difference in the amounts of Fub1p and Bml10p was observed between the experimental groups as indicated by the label-free quantification (LFQ) method (Fig. 4, Table S2), suggesting that they are not associated with glucose restriction-induced differences in 20S proteasome activity. The 14 subunits that constitute the 20S proteasome were detected in all analyzed samples (Table S1). The quantitative analysis of proteasomal subunits revealed that glucose restriction does not interfere in the stoichiometry of proteasomal components since the amount of each of the 14 subunits was identical when glucose restriction and control samples were compared (Fig. S3). Interestingly, mass spectrometry analysis revealed two consistently phosphorylated sites: Serine 13 in subunit α2 and Threonine 55/Serine 56 in subunit α5 (Table S3). While the first one was present in both control and glucose restricted proteasomes, the phosphopeptide Thr55/Ser56 was detected in only one control sample, while in glucose restricted samples, it was detected in 3 out of 4 samples (Fig. 5, Table S3). It is important to note that the phosphopeptide has 2 residues prone to phosphorylation (threonine 55 and serine 56) located side-by-side in the primary sequence (Fig. 5, Table S3, Suppl. Data 1). Interestingly, the residues are located within a flexible region of α5 proteasome subunit, accessible to the cytoplasm (Fig. 5 E), suggesting that Thr55/Ser56 may be a regulatory site. Unfortunately, we were not able to detect ions y11 and y12 (Suppl. data 1), which hindered the precise identification of the residue bearing the phosphoryl group.

### Phosphorylation of threonine 55 in α5-subunit facilitates the interaction between α-synuclein and proteasome α ring

**Figure 6:**
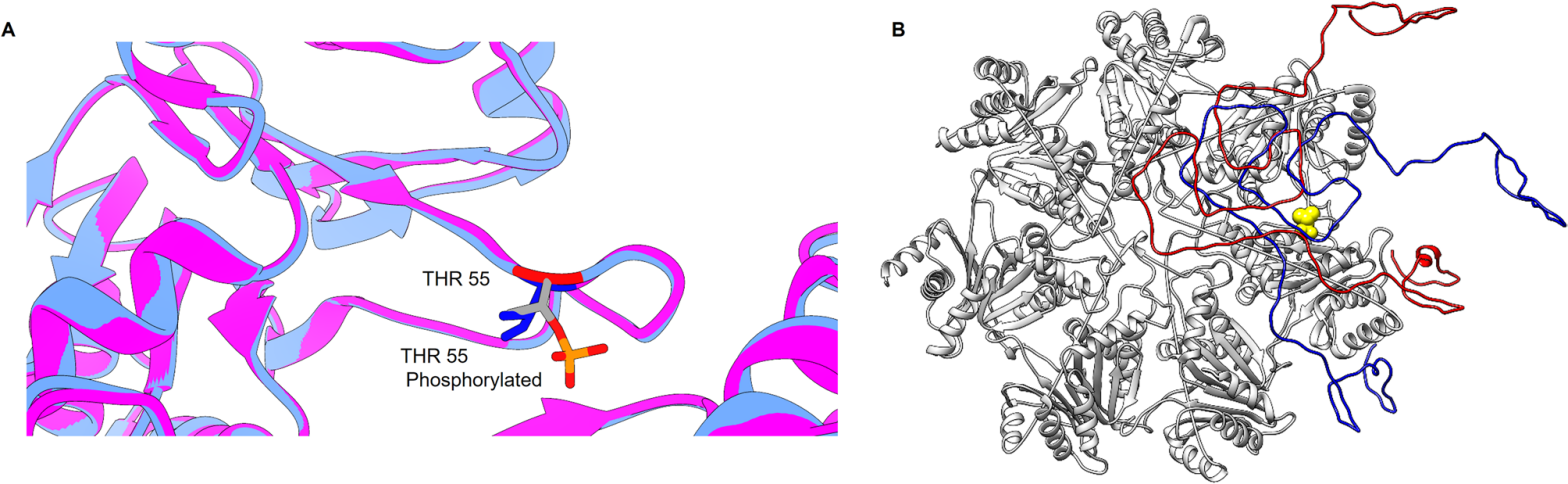
Phosphorylation of Thr55 does not change local conformation but may affect the interaction between α5-subunit and proteins. (**A**) Representation of part of the three-dimensional structure of the 20S proteasome (pdb 3D29) focused on the region where the Thr55 residue susceptible to phosphorylation is located. The structure with dephosphorylated Thr55 is shown in pink while the simulated structure with phospho-Thr55 in represented in blue. (**B**) Representation of models of interaction between the 20S proteasome α-ring and α-synuclein. The models were generated with the Haddock program. Interaction of α-synuclein with the proteasome α-ring in the absence or presence of Thr55 phosphorylation is represented in blue and red, respectively. Phosphorylated threonine 55 is shown in yellow.

To gain further insight into the impact of α5-subunit phosphorylation on protein degradation, the interaction of α-synuclein protein with the proteasome α ring was modeled in silico using a docking approach^42^. The model was generated considering threonine 55 as the phosphorylated residue. Although mass spectrometry data did not allow the discrimination between threonine 55 or serine 56 as the phosphoryl bearing residue, data on the literature suggest that threonine 55 is phosphorylated in the human cell lineage MCF7 after starvation or rapamycin-induced autophagy^43^. Since our experimental paradigm involves energy restriction, it is possible that threonine 55 is the residue phosphorylated in glucose restriction samples. The models obtained in the presence or absence of phospho-threonine 55 are superposable, indicating that the modification does not significantly impact the conformation of the surrounding region (Fig. 6A). Interestingly, the interaction of the proteasomal α ring with the protein α-synuclein is predicted to be increased in extent by Thr55 phosphorylation, positioning α-synuclein closer to the entrance pore in comparison with the model with non-phosphorylated Thr55 (Fig. 6B, Table 1). In addition, the reduced cluster size, root-mean-square deviation of atomic positions (RMSD) and z-score parameters for the glucose restriction model suggest that the interaction between 20S proteasome and α-synuclein is more favorable in this model when compared with the control one (Table 1).

**Table 1:** High ambiguity driven protein–protein docking (HADDOCK) modeling results.

	Control	Glucose restriction
HADDOCK score	301.5 ± 11.5	295.5 ± 41.5
Cluster size	8	4
root-mean-square deviation of atomic positions (RMSD) for the overall lowest energy structure	7.2 ± 0.0	0.9 ± 0.5
Van der Waals energy	− 72.2 ± 1.9	− 70 ± 16.5
Electrostatic energy	− 738.5 ± 89.0	− 722.2 ± 87.8
Z-score	− 1.6	− 2.1

Control: proteasome α-ring devoid of phosphorylation; Glucose restriction: proteasome α-ring with the α5-subunit phosphorylated at Thr55.

## Discussion

Given the importance of proteasome activity for cellular homeostasis and the stimulation of the activity of this proteolytic complex by glucose restriction, this work focused on a better understanding of the effects of glucose restriction on the *S. cerevisiae* proteasome. We previously showed that cell extracts obtained from *S. cerevisiae* cells cultured under glucose restriction had increased proteasome activity^18^. The results indicated that the increase in activity was not the result of higher amounts of proteasome in glucose restricted-cells, but an increase in the coupling of the 20S core to the 19S regulatory subunit would increase proteasome activity^44^ and was not investigated in our previous work. Herein, we fill this gap by showing that glucose restriction-induced increase in proteasome activity is not due to increased coupling between the 20S catalytic core and the 19S regulatory unit. The effect of the intervention could be indirect, through labile and transient interactions between the 20S proteasome and proteins present in the cellular extract. We observed that proteasomes purified from yeast cells cultured under glucose restriction present site-specific increased activity. Indeed, even after the purification process, which is long and stringent, proteasomes isolated from glucose restricted cells presented increased chymotrypsin-like activity, suggesting that glucose restriction promotes robust and lasting changes on the 20S proteasome particle. It should be noted that labile interactions promoted by glucose restriction cannot be ruled out, since the difference in activity between the experimental groups is smaller in isolated proteasomes when compared to the difference observed in cell extracts. It is also possible that during the purification process the proteasome particles lose post-translational modifications, attenuating the difference between the experimental groups.

Glucose restriction-induced increase in chymotrypsin-like activity translated into increased proteolytic capacity, since glucose restricted-proteasomes were more efficient at degrading β-casein and α-synuclein. This increased proteolytic capacity does not seem to extend to all 20S substrate proteins, since it was not observed when glutaredoxin-2 was used as substrate. This finding is important since degradation rates of fluorogenic substrates do not always accurately represent proteasome degradation capacity of whole proteins^33^, which are the actual proteasome substrates in the cellular context. Taken together, the data indicate that glucose restriction promotes robust and long-lasting changes in the 20S proteasome leading to increased chymotrypsin-like peptidase activity and proteolytic capacity, suggesting that glucose restriction can impact on proteasomal degradation of proteins in vivo.

Since we found higher proteolytic activity in proteasomes isolated from glucose restricted cells, we went on to investigate whether culturing cells under glucose restriction promoted modifications on the catalytic core that could be associated with increased activity. A number of works show that mammalian 20 and 26S proteasomes undergo post translational modifications, including phosphorylation, that alter their activity and/or substrate specificity^45^ for review see^9,46^. Similarly to mammalian proteasomes, the 20S proteasome of *Saccharomyces cerevisiae* has several predicted post-translational modification sites^9,15^. Some of them have already been confirmed, such as the S-glutathionylation of the cysteine residues 76 and 221 in the α-5 subunit^17^, which modifies proteasomal gating^17^, and the phosphorylation of residues in the α1, α2, α3, α4 and α7 subunits^47–50^ for review see^15^. While the effect of S-glutathiolylation on proteasome activity is well characterized^17,51,52^ the same is not true for many of the phosphorylated sites. Interestingly, the electrophoretic separation of proteasomes purified from glucose restricted or control cells in 2 dimensional gels suggested a number of glucose restriction-induced differences in isoelectric points of specific proteasomal subunits. The analysis of samples by mass spectrometry revealed only 2 phosphorylated sites, despite the spots with altered isoelectric point observed in 2D electrophoresis gels and several phosphorylated sites already described for the 20S proteasome^15^. This discrepancy may be explained by the fact that the conditions used for proteasome purification and sample preparation were not ideal for phosphopeptide detection. Indeed, no phosphatase inhibitor or phosphopeptide enrichment strategies were used, which certainly contributed to the low detection of phosphorylated residues^53^. However, even under the unfavorable conditions used in this work, the phospho-Thr55/Ser56 in α5-subunit was detected in 3 out of 4 glucose restricted samples suggesting that many proteasome particles present in the glucose restricted samples might be phosphorylated at this site. It is interesting to note that glutathionylation of cysteine residues of α5-subunit was reported to alter the 20S gate opening^17^, suggesting that α5-subunit is a hotspot for post-translational regulation of the 20S proteasome activity.

Threonine 55 and Serine 56 from α5-subunit were already reported to be phosphorylated in human lymphoblasts subjected to genotoxic damage induced by doxorubicin, a DNA intercalator^16^. The authors showed that doxorubicin treatment increased the chymotrypsin-like and post-acidic activities of the 20S proteasome and induced the phosphorylation of Thr55 and Ser56 on the α5-subunit, but did not establish a causal relationship between phosphorylation and activity. Thr55 was also reported to be phosphorylated in response to rapamycin treatment or amino acid deprivation in a human epithelial cell line^43^, suggesting that this modification is part of the autophagic signaling. The authors, however, did not assess proteasome activity. It is tempting to speculate that Thr55 phosphorylation in response to nutritional stress is conserved from yeast to humans since we α5 Thr55 may also be phosphorylated in our glucose restricted samples.

Phosphorylation sites are often described in flexible regions of proteins^54^. This is fundamental for the insertion of negative charges, through phosphorylation, to impact the protein structure and, consequently, its function^54^. Threonine 55 and serine 56 are located in a flexible region of the α5-subunit which is also exposed to the solvent. It is thus possible that a conformational change takes place in the α5-subunit upon Thr55/Ser56 phosphorylation. In addition, our results demonstrate that glucose restriction increased peptidase activity of the 20S proteasome in a site-specific manner, suggesting that the changes promoted by this experimental intervention on the 20S proteasome are subtle. Site-specific changes in proteasome activity have already been described in the literature. Several ligands were reported to interact at sites distant from the catalytic threonine residues, regulating the proteolytic activity through allosteric modulation of the complex. For example, a set of peptides rich in proline and arginine were shown to bind pockets between the α5 and α6 subunits inhibiting or activating the 20S proteasome depending on its amino acid sequence^55^. The coupling of regulatory subunits to the 20S catalytic complex has also been described as capable of allosterically regulating the catalytic core. Indeed, the coupling of the PA200 subunit with the 20S proteasome in mammalian cells induces a change in the proteasome's conformation that leads to an enlargement of the region where the β2 subunit is located and a narrowing of the region where the β5 and β1 subunits are found. This conformational change results in increase of trypsin-like activity and inhibition of chymotrypsin-like and post-acidic-like activities^52,56^. To determine whether glucose restriction-induced increase in proteasome activity was accompanied by changes in the particle spatial conformation, we analyzed the structure of purified proteasome particles using different approaches. The results obtained from cryo-TEM, SAXS or circular dichroism did not reveal differences between glucose restricted and control groups, indicating that glucose restriction in general and Thr55/Ser56 phosphorylation in particular do not affect the opening of the 20S proteasome gate or induce other important conformational changes in the particle.

Even without promoting important changes in the spatial conformation of the proteasome, Thr55/Ser56 phosphorylation may still influence proteasome interaction with protein substrates, subsequently impacting on their degradation. Experimental data show that when phosphorylation occurs in a residue located at interaction interfaces, it may directly modulate the strength of the protein–protein interaction^57^. Contributing to this idea, it was reported in Jukart cells that phosphorylation of Tyr-950 of the 19S RPN2 subunit enhances RPN13 subunit binding through specific interactions with positively charged residues in RPN13, without inducing conformational changes in RPN2^58^. The docking modeling of the interaction between the proteasome α-ring and α-synuclein performed in this work suggests that the presence of a phosphoryl group in Thr55 enhances the interaction of α-synuclein with the α-ring. In fact, although the energy of van der Waals and electrostatic ​​calculated for each group were similar, the cluster size and root-mean-square deviation of atomic positions (RMSD) parameter were smaller for the glucose restricted group, indicating a more stable interaction between proteasome α-ring and α-synuclein when the Thr55 is phosphorylated. Since 20S proteasomes isolated from glucose restricted cells were more efficient at degrading α-synuclein compared with the ones isolated from control cells, it is tempting to speculate that stabilizing the interaction between the 20S proteasome α-ring and α-synuclein leads to increased protein degradation.

It is interesting to mention that the differences on proteasome activity observed between glucose restriction and control groups were analyzed after 7.5 h of culture, during logarithmic phase of growth, indicating that glucose restriction effects are not induced by glucose exhaustion. While not the focus of the present work, it is tempting to speculate that the conditions used in the pre-inoculums may have induced epigenetic marks in mother cells which would be transmitted to their daughters, ultimately impacting on proteasome activity or sensitizing the progeny in such a way that proteasome activity was higher, even in the presence of glucose^59,60^. Also, the cells may have responded to glucose concentration per se. Indeed, it was reported that Snf1 (the yeast homologue of AMPK) monitors glucose concentration changes and absolute levels, influencing Mig1 repressor cellular localization^61,62^. The fact that *S. cerevisiae* can sense absolute glucose levels and changes in concentration may also contribute to altered proteasome activity in the presence of glucose, since glucose concentration of GR and C is significantly different.

The data obtained in this work indicate that glucose restriction increases chymotrypsin-like activity as well as the proteolytic capacity of the 20S proteasome purified from *S. cerevisiae*. The increased proteolytic capacity may be related, at least in part, to glucose restriction-induced Thr55/Ser56 phosphorylation at the α5-subunit. In addition, glucose-restriction effects on the 20S proteasome do not appear to involve significant changes in the particle’s structure. It will be interesting to know, in the future, whether the phosphorylation in the α5-subunit occurs in Thr55, Ser 56 or both and whether there is a causal relationship between the phosphorylated site and increased proteolysis.

## Methods

### Cell strain and culture

*S. cerevisiae* from the strain RJD1144^63^ was cultured under continuous shaking at 160 rpm, 30 °C, in liquid YP (1% yeast extract, 2% peptone) containing 2.0% or 0.5% glucose for control (C) or glucose restriction (GR) conditions, respectively. Pre-inoculums grown for 16 h under C or GR conditions were used start the assay cultures. The inoculums were made by inoculating the cells in the pre-inoculum to the optical density of 0.1 at 600 nm in new Erlenmeyer flasks containing C or GR media. Cells were harvested after 7.5 h of growth, time at which both experimental groups were in the mid-log phase of growth (Supplementary Fig. S4).

### 20S proteasome purification

Cells from 3 L of culture for each experimental condition were harvested by centrifugation (1200×*g*, 6 min, 4 °C). All purifications were carried out in the absence of ATP regenerating system. The cell pellets were washed in water, resuspended in 15 mL of buffer 1 (for buffer composition see Table 2) and lysed in a bead beater (BioSpec, Oklahoma, USA) using 7.5 mL of glass beads, through 12 cycles of 20 s agitation and 40 s pause on ice, to avoid sample overheating. The obtained extracts were centrifuged (10,000×*g*, 30 min, 4 °C) and the supernatant was used in subsequent steps. The cellular extract was subjected to a nickel affinity chromatography using a 12 mL column filled with Ni NTA Agarose (Qiagen, Venlo, Netherlands). The chromatographic separation was performed in a 20 to 400 mM imidazole linear gradient using buffers 1 and 2 (Table 2). One mL fractions were automatically collected and monitored for protein presence by absorbance at 280 nm in an Äkta purifier (GE Healthcare, Uppsala, Sweden). Protein-containing fractions were assayed for proteasome activity using the fluorogenic peptide *suc-*LLVY-AMC, and the ones positive for proteasome activity were pooled and desalted in a 5 mL HiTrap® desalt column (GE Healthcare, Uppsala, Sweden) using buffer 3 (Table 2). Finally, samples were subjected to anion exchange chromatography performed in a 5 mL HiTrap® Q column (GE Healthcare, Uppsala, Sweden) using buffers 3 and 4 (Table 2). Fractions positive for proteasome activity were pooled and subjected to buffer exchange (buffer 5) and concentration using 100 kDa Amicon® Ultra centrifugal filters (Merck Millipore, Massachusetts, EUA). Protein concentration in samples was determined by the Bradford method^64^ and sample purity was verified by SDS-PAGE (Supplementary Fig. S2).

**Table 2:** Buffers used in proteasome purification and enzymatic assays.

Buffer number	Composition (mM)					
	Tris–Cl pH 7.5	Hepes pH 7.2	MgCl_2_	NaCl	KCl	Imidazole
1	50	–	5	500	20	20
2	50	–	5	500	20	400
3	–	10	5	–	100	–
4	–	10	5	–	1000	–
5	50	–	5	–	–	–

### Proteasome peptidase activity and coupling

One µg of purified proteasomes was incubated in buffer 5 (Table 2) in the presence of 125 µM S*uc*-LLVY-AMC, 200 µM Z-LLE-AMC or 200 µM Z-ARR-AMC for assessing chymotrypsin-, caspase- or trypsin-like activities, respectively. Fluorescence increase, indicative of peptide cleavage, was followed for 30 min at 30 °C in a Flex Station 3 (Molecular Devices, California, EUA) plate reader using excitation ʎ 365 nm and emission ʎ 440 nm. For proteasome activity detection in chromatographic fractions, 10 µL aliquots were incubated with 50 µM S*uc*-LLVY-AMC and the increase in fluorescence was followed as described above. Finally, the evaluation of proteasome activity in cellular extracts was performed by incubating 50 µg protein extract with 125 µM S*uc*-LLVY-AMC and following the increase in fluorescence.

To analyze the coupling of the 20S core to the 19S regulatory unit, cellular protein was submitted to separation in native gel electrophoresis. The experiment was performed according to the protocol described elsewhere^65^. Thirty µg protein from cellular extract were applied to the gel. The gel was run for 4 h, at 150 V and 4 °C, and stained with Coomassie Blue. The bands corresponding to the 20S and 26S particles were visualized by in-gel activity by the spreading of 100 µM S*uc*-LLVY-AMC onto the gel.

### 20S proteasome proteolytic activity

The proteolytic capacity of purified 20S proteasomes was assessed according to^17^, using β-casein, α-synuclein and glutaredoxin-2 as substrate, since these proteins were shown to be degraded by the 20S proteasome^34–36,66^. Briefly, 5 µg of purified proteasomes were incubated with 20 µg of β-casein for 15 min at 30 °C; 10 µg of α-synuclein or glutaredoxin-2 for 90 min at 37 °C. In assays involving β-casein, proteasome particles were removed from the mixture by centrifugation using 100 kDa Amicon® Ultra Centrifugal filters (Merck Millipore, Massachusetts, EUA). In assays involving α-synuclein or glutaredoxin-2, the whole sample was mixed to sample buffer (100 mM Tris–Cl pH 6.8, 10% glycerol, 1% SDS, 0.02% bromophenol blue), boiled for 5 min and subjected to SDS-PAGE. Casein filtered samples were also mixed to sample buffer and boiled for 5 min before being submitted to SDS-PAGE. The same amounts of proteins were incubated in the absence of the proteasome and considered as control. The gels were subsequently stained with Coomassie Brilliant blue.

### Cryo transmission electron microscopy of isolated 20S proteasomes

Proteasomes were deposited onto a Quantifoil R2/2 holey carbon grid (Quantifoil, Jena, Germany) ionized by glow discharge. The grid was blotted with a filter paper for 2 s and directly plunged into liquid ethane cooled down by liquid nitrogen, using a FEI Vitrobot Mark IV (Thermo Fisher Scientific, Massachusetts, USA) operated at 22 °C and 100% relative humidity. The cryo-specimens were transferred into a Gatan 626 cryo-holder and observed at -180 °C in a JEOL 2010F Cryo-Transmission Electron Microscope (JEOL, Tokyo, Japan) equipped with a field emission gun and operated at 200 kV. Images were recorded with a 4 K Gatan Ultrascan 1000 camera (Gatan, Pleasanton, USA) with − 2 μm of nominal defocus on the camera (pixel size 0.29 or 0.236 nm) at a nominal magnification of 60,000 × under low electron-dose conditions, and analyzed with the software CryoSPARC^67^.

### Small angle X-ray scattering analysis of isolated 20S proteasomes

SAXS measurements were performed on a laboratory based SAXS system Bruker-Nanostar (Bruker-Nanostar, Karlsruhe, Germany), equipped with a Cu microfocus source Genix3D (λ_Cukα_ = 1,54 Å), focusing mirrors Fox3D and two sets of scatterless slits, all provided by Xenocs (Xenocs, Grenoble, France). Approximately 100 µL of purified proteasomes at 0.7 µg/µL were placed into reusable sample holders which allows the acquisition of sample and blank in the same conditions, which is very important for a proper data treatment. The sample to detector distance was 660 mm and the 2D SAXS images were collected on a Bruker-Vantec2000 detector. Azimuthal integrations were performed on the software present on the equipment, providing 1D SAXS curves of the intensity as a function of the reciprocal space momentum transfer modulus $q$, defined as $q=4\pi sin\theta /\lambda$, were $2\theta$ is the scattering angle. 24 frames of 1800s were collected for each sample in order to check sample stability during the measurement. Data treatment were performed using the program package SUPERSAXS package (Oliveira and Pedersen, unpublished; http://portal.if.usp.br/gfcx/pt-br/node/354) and the program WIFT program^68^ was used to determine the pair distance distribution function (p(*r*))^69^. Further modeling of the scattering data was performed using a form factor of a hollow cylinder (equation described in^17^), with the parameters outer radius (Rout), inner radius (Rin), and length (L).

### Circular dichroism analysis of isolated proteasomes

CD spectra were collected using a Jasco 810 spectropolarimeter (JASCO International Co., Ltd., Tokyo, Japan) saturated with nitrogen and operating at room temperature. Proteasome solutions (approximately 0.2 µg/µL) were loaded into quartz cuvettes (1 mm pathlength) and data were recorded from 190 to 260 nm at 100 nm/min with a wavelength step of 0.5 nm. Only data with an absorbance (Abs) < 1.5 were used for analysis. The resulting spectra were obtained by averaging five accumulations, subtracting background using buffer measurements, and applying 7-point fast Fourier transform (FFT) filters to eliminate noise. Ellipticity units were calculated using mean residue weight, optical pathlength, and proteasome concentration.

### Bi-dimensional gel electrophoresis

One hundred and fifty µg of purified 20S proteasome were diluted in rehydration buffer (8 M urea, 2% CHAPS, 0.5% Pharmalyte® and 0,007% bromophenol blue) and applied onto 18 cm IPG strips (pH 4–7) (GE Healthcare, Uppsala, Sweden). After overnight rehydration at room temperature, the sample proteins were separated according to their isoelectric point in a IPGphor 3 (GE Healthcare, Uppsala, Sweden) system, running under the parameters listed in the manufacturer's manual. The IPG strips were equilibrated in equilibration buffer (50 mM Tris–Cl pH 6.8, 6 M urea, 30% glycerol and 1% SDS), placed on the top of a 12,5% acrylamide gel and separated according to their mass in a vertical electrophoresis system (GE Healthcare, Uppsala, Sweden). The gels were stained with colloidal Coomassie blue G-250, scanned in a ChemiDoc MP imaging system (Bio-Rad Lab., California, USA), and the migration pattern between C or GR samples was compared using ImageMaster 2D Platinum software (GE Healthcare, Uppsala, Sweden).

### Mass spectrometry of isolated proteasomes

Ten µg of purified proteasomes were digested by sequencing grade modified trypsin (Promega), after previous sample reduction (10 mM dithiothreitol, 0.1% sodium deoxycholate, 1 h at 37 °C) and alkylation (15 mM iodoacetamide, 30 min, 37 °C). Trypsin digestion was carried out for 4 h at 37 °C by using a protein to trypsin ratio of 1:40. Proteasomes were further digested overnight at 37 °C after a second addition of trypsin in the ratio of 1:50. The samples were acidified with 0.5% v/v trifluoracetic acid (TFA) and centrifuged at 14.000 rpm, 30 min, 4 °C. The supernatant was subsequently desalted using in house-built C18-Stage Tips, following the protocol described by^70^. Tryptic peptides were eluted with acetonitrile:water:TFA (60:39.9:0.1), vacuum dried and resuspended in 0.1% TFA in water. After a final centrifugation step (14.000 rpm, 30 min, 4 °C), the supernatant was transferred to an injection plate of a nano-LC Easy-nano LC™ 1200 (Thermo Fisher Scientific, Massachusetts, USA) system. The samples were loaded onto an Acclaim™ PepMap™ 100 C18 trap column (3 µm particle size, 75 µm diameter, 2 cm length; Thermo Fisher Scientific, Massachusetts, USA), washed with 20 µL of solvent A (0.1% TFA in water) and eluted at a flow of 300 nL/min onto an Acclaim™ PepMap™ 100 C18 analytical column (2 µm particle size, 50 µm diameter, 15 cm length; Thermo Fisher Scientific, Massachusetts, USA). For nano-LC, mobile phase A consisted of 0.1% TFA in water, while mobile phase B consisted of acetonitrile:water:TFA (80:19.9:0.1). The linear gradient used in nano-LC was: 5% to 28% of B over the first 80 min followed by a linear gradient of 28% to 40% in 10 min. A third linear gradient of 40% to 95% of B in 2 min was applied and held constant during 12 additional min, totalizing 104 min of run. Data was acquired in an Orbitrap Fusion Lumos® (Thermo Fisher Scientific, Massachusetts, USA) mass spectrometer equipped with a nano electrospray ion source (Thermo Fisher Scientific, Massachusetts, USA, using a full scan (*m/z* range 400 to 1600) followed by data dependent MS2 scans (*m/z* range 100 to 2000), in a cycle time of 3 s, using an isolation window of 1.2 Th and collision energy of 30. Selected precursor ions were excluded for 40 s. MS/MS spectra were searched using MaxQuant software (Version 1.6.2.10)^71^ and Andromeda search engine (August, 2018)^72^. Peptides were matched against the Uniprot database for *Saccharomyces cerevisiae* database (6721 entries in Nov 10th, 2019), with fixed Cys carbamidomethylation and variable Met oxidation modification, Ser, Thr and Tyr phosphorylation and N-terminal acetylation. MaxQuant results were further analyzed using Perseus software^73^. One missed cleavage was considered for the analysis. The mass tolerance for precursor ions was 4.5 ppm and for fragment ions, 20 ppm. Common contaminants were identified and excluded of the final analysis and the FDR for proteins and peptides was set at 0.01. Other parameters were kept as default.

### In silico modeling of *α*-synuclein interaction with the 20S proteasome α ring

A molecular docking strategy was applied using the software HADDOCK (High Ambiguity Driven protein − protein Docking)^74^. The modeling was performed using the crystallographic model (PDB entry 3D29) of the seven α subunits that compose the 20S proteasome α ring. The α-ring was fixed in space and two different conditions were compared: the control condition, in which no sites were considered phosphorylated, and GR condition, in which the residue of threonine 55 in the α-5 subunit was considered phosphorylated. The monomer of α-synuclein with PDB entry 2N0A was used to evaluate the impact of the Thr55 phosphorylation on the interaction between the proteasome α-ring and α-synuclein.

### Supplementary Information

Supplementary Information 1.

Supplementary Information 2.

Supplementary Information 3.

Supplementary Information 4.

Supplementary Information 5.

Supplementary Information 6.

## Data Availability

All data generated or analyzed during this study are included in this published article (and its Supplementary Information files).
