# Supplementary material for: Glucose restriction in Saccharomyces cerevisiae modulates the phosphorylation pattern of the 20S proteasome and increases its activity
Source: Sci Rep. 2023 Nov 8;13:19383. doi: 10.1038/s41598-023-46614-x (PMC10632367; doi:10.1038/s41598-023-46614-x)
Supplement: Supplementary file 1 — Supplementary Information 1. [file 41598_2023_46614_MOESM1_ESM.docx]

Supplementary Data 1 – Simulated fragmentation of the peptide **ATSPLLESDSIEK**.

Simulated fragmentation pattern for **unmodified** peptide **ATSPLLESDSIEK**.

| **b** |  |  |  | **y** | **y^+2^** |
| --- | --- | --- | --- | --- | --- |
|  |  |  |  |  |  |
| --- | 1 | **A** | 13 | --- | --- |
| 173.0921 | 2 | **T** | 12 | 1318.6737 | 659.8405 |
| 260.1241 | 3 | **S** | 11 | 1217.6260 | 609.3166 |
| 357.1769 | 4 | **P** | 10 | 1130.5939 | 565.8006 |
| 470.2609 | 5 | **L** | 9 | 1033.5412 | 517.2742 |
| 583.3450 | 6 | **L** | 8 | 920.4571 | 460.7322 |
| 712.3876 | 7 | **E** | 7 | 807.3731 | 404.1902 |
| 799.4196 | 8 | **S** | 6 | 678.3305 | 339.6689 |
| 914.4466 | 9 | **D** | 5 | 591.2984 | 296.1529 |
| 1001.4786 | 10 | **S** | 4 | 476.2715 | 238.6394 |
| 1114.5626 | 11 | **I** | 3 | 389.2395 | 195.1234 |
| 1243.6052 | 12 | **E** | 2 | 276.1554 | 138.5813 |
| --- | 13 | **K** | 1 | 147.1128 | 74.0600 |

Elemental Composition: [C59 H101 N14 O24](https://prospector.ucsf.edu/prospector/cgi-bin/mssearch.cgi?search_name=msisotope&output_type=HTML&report_title=MS-Isotope&display_graph=1&version=6.4.5&distribution_type=Elemental%20Composition&instrument_name=ESI-Q-high-res&parent_charge=3&elemental_composition=C59%20H101%20N14%20O24&)

| **MH^+1^(av)** | **MH^+1^(mono)** | **MH^+2^(av)** | **MH^+2^(mono)** | **MH^+3^(av)** | **MH^+3^(mono)** |
| --- | --- | --- | --- | --- | --- |
| 1390.5394 | 1389.7108 | 695.7734 | 695.3590 | 464.1848 | 463.9084 |
|  |  |  |  |  |  |

Simulated fragmentation pattern for peptide **ATSPLLESDSIEK phopshorilated (∆m = 79.966 Da) at position 2**.

| **b-H_3_PO_4_** | **b** |  |  |  | **y** | **y^+2^** | **y-H_3_PO_4_** | **y-H_3_PO_4_^+2^** |
| --- | --- | --- | --- | --- | --- | --- | --- | --- |
|  |  |  |  |  |  |  |  |  |
| --- | --- | 1 | **A** | 13 | --- | --- | --- | --- |
| 155.0815 | 253.0584 | 2 | **T(phospho)** | 12 | 1398.6400 | 699.8236 | 1300.6631 | 650.8352 |
| 242.1135 | 340.0904 | 3 | **S** | 11 | 1217.6260 | 609.3166 | --- | --- |
| 339.1663 | 437.1432 | 4 | **P** | 10 | 1130.5939 | 565.8006 | --- | --- |
| 452.2504 | 550.2273 | 5 | **L** | 9 | 1033.5412 | 517.2742 | --- | --- |
| 565.3344 | 663.3113 | 6 | **L** | 8 | 920.4571 | 460.7322 | --- | --- |
| 694.3770 | 792.3539 | 7 | **E** | 7 | 807.3731 | 404.1902 | --- | --- |
| 781.4090 | 879.3859 | 8 | **S** | 6 | 678.3305 | 339.6689 | --- | --- |
| 896.4360 | 994.4129 | 9 | **D** | 5 | 591.2984 | 296.1529 | --- | --- |
| 983.4680 | 1081.4449 | 10 | **S** | 4 | 476.2715 | 238.6394 | --- | --- |
| 1096.5521 | 1194.5290 | 11 | **I** | 3 | 389.2395 | 195.1234 | --- | --- |
| 1225.5947 | 1323.5716 | 12 | **E** | 2 | 276.1554 | 138.5813 | --- | --- |
| --- | --- | 13 | **K** | 1 | 147.1128 | 74.0600 | --- | --- |

Elemental Composition: [C59 H102 N14 O27 P1](https://prospector.ucsf.edu/prospector/cgi-bin/mssearch.cgi?search_name=msisotope&output_type=HTML&report_title=MS-Isotope&display_graph=1&version=6.4.5&distribution_type=Elemental%20Composition&instrument_name=ESI-Q-high-res&parent_charge=3&elemental_composition=C59%20H102%20N14%20O27%20P1&)

| **MH^+1^(av)** | **MH^+1^(mono)** | **MH^+2^(av)** | **MH^+2^(mono)** | **MH^+3^(av)** | **MH^+3^(mono)** |
| --- | --- | --- | --- | --- | --- |
| 1470.5193 | 1469.6771 | 735.7633 | **735.3422** | 490.8447 | 490.5639 |

Simulated fragmentation pattern for peptide **ATSPLLESDSIEK phopshorilated (∆m = 79.966 Da) at position 3**.

| **b-H_3_PO_4_** | **b** |  |  |  | **y** | **y^+2^** | **y-H_3_PO_4_** | **y-H_3_PO_4_^+2^** |
| --- | --- | --- | --- | --- | --- | --- | --- | --- |
|  |  |  |  |  |  |  |  |  |
| --- | --- | 1 | **A** | 13 | --- | --- | --- | --- |
| --- | 173.0921 | 2 | **T** | 12 | 1398.6400 | 699.8236 | 1300.6631 | 650.8352 |
| 242.1135 | 340.0904 | 3 | **S(phospho)** | 11 | 1297.5923 | 649.2998 | 1199.6154 | 600.3113 |
| 339.1663 | 437.1432 | 4 | **P** | 10 | 1130.5939 | 565.8006 | --- | --- |
| 452.2504 | 550.2273 | 5 | **L** | 9 | 1033.5412 | 517.2742 | --- | --- |
| 565.3344 | 663.3113 | 6 | **L** | 8 | 920.4571 | 460.7322 | --- | --- |
| 694.3770 | 792.3539 | 7 | **E** | 7 | 807.3731 | 404.1902 | --- | --- |
| 781.4090 | 879.3859 | 8 | **S** | 6 | 678.3305 | 339.6689 | --- | --- |
| 896.4360 | 994.4129 | 9 | **D** | 5 | 591.2984 | 296.1529 | --- | --- |
| 983.4680 | 1081.4449 | 10 | **S** | 4 | 476.2715 | 238.6394 | --- | --- |
| 1096.5521 | 1194.5290 | 11 | **I** | 3 | 389.2395 | 195.1234 | --- | --- |
| 1225.5947 | 1323.5716 | 12 | **E** | 2 | 276.1554 | 138.5813 | --- | --- |
| --- | --- | 13 | **K** | 1 | 147.1128 | 74.0600 | --- | --- |
|  |  |  |  |  |  |  |  |  |

Elemental Composition: [C59 H102 N14 O27 P1](https://prospector.ucsf.edu/prospector/cgi-bin/mssearch.cgi?search_name=msisotope&output_type=HTML&report_title=MS-Isotope&display_graph=1&version=6.4.5&distribution_type=Elemental%20Composition&instrument_name=ESI-Q-high-res&parent_charge=3&elemental_composition=C59%20H102%20N14%20O27%20P1&)

| **MH^+1^(av)** | **MH^+1^(mono)** | **MH^+2^(av)** | **MH^+2^(mono)** | **MH^+3^(av)** | **MH^+3^(mono)** |
| --- | --- | --- | --- | --- | --- |
| 1470.5193 | 1469.6771 | 735.7633 | **735.3422** | 490.8447 | 490.5639 |
