# Supplementary material for: Glucose restriction in Saccharomyces cerevisiae modulates the phosphorylation pattern of the 20S proteasome and increases its activity
Source: Sci Rep. 2023 Nov 8;13:19383. doi: 10.1038/s41598-023-46614-x (PMC10632367; doi:10.1038/s41598-023-46614-x)
Supplement: Supplementary file 2 — Supplementary Information 2. [file 41598_2023_46614_MOESM2_ESM.docx]

**
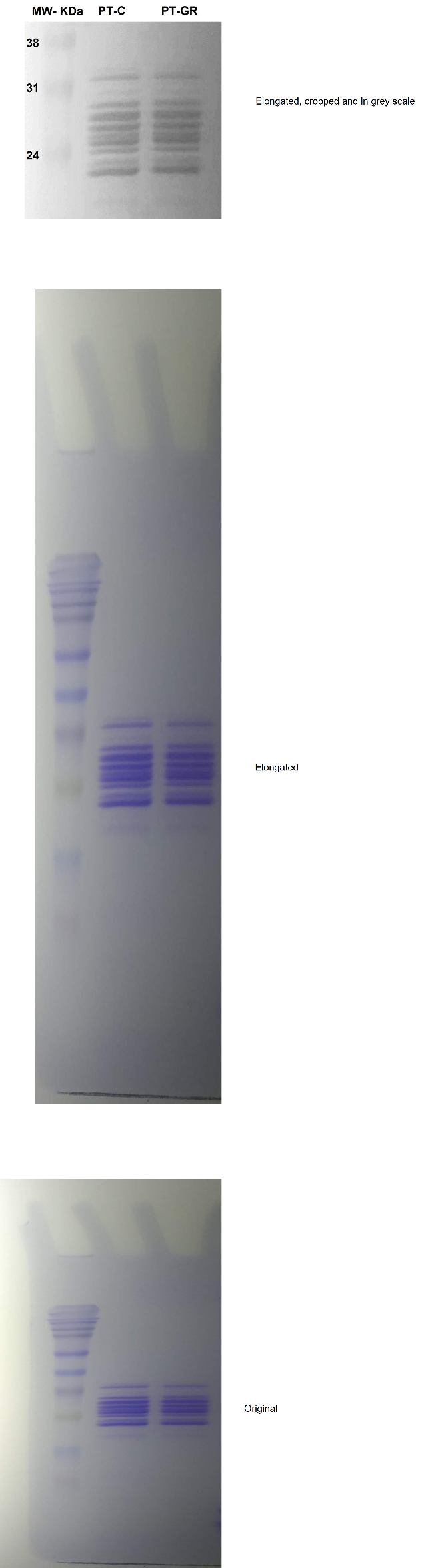
Supplementary Figure 1**

Supplementary figure 1: SDS-PAGE of 20S proteasome purified from cells grown under control (PT-C) or glucose restriction (PT-GR) condition. Five µg of purified proteasomes were submitted to SDS-PAGE and the gel was stained with Coomassie brilliant blue as described in the Materials and experimental procedures section. The original image was elongated, cropped and changed for the grey scale to facilitate proteasome subunit visualization.

**Supplementary Figure 2**

**
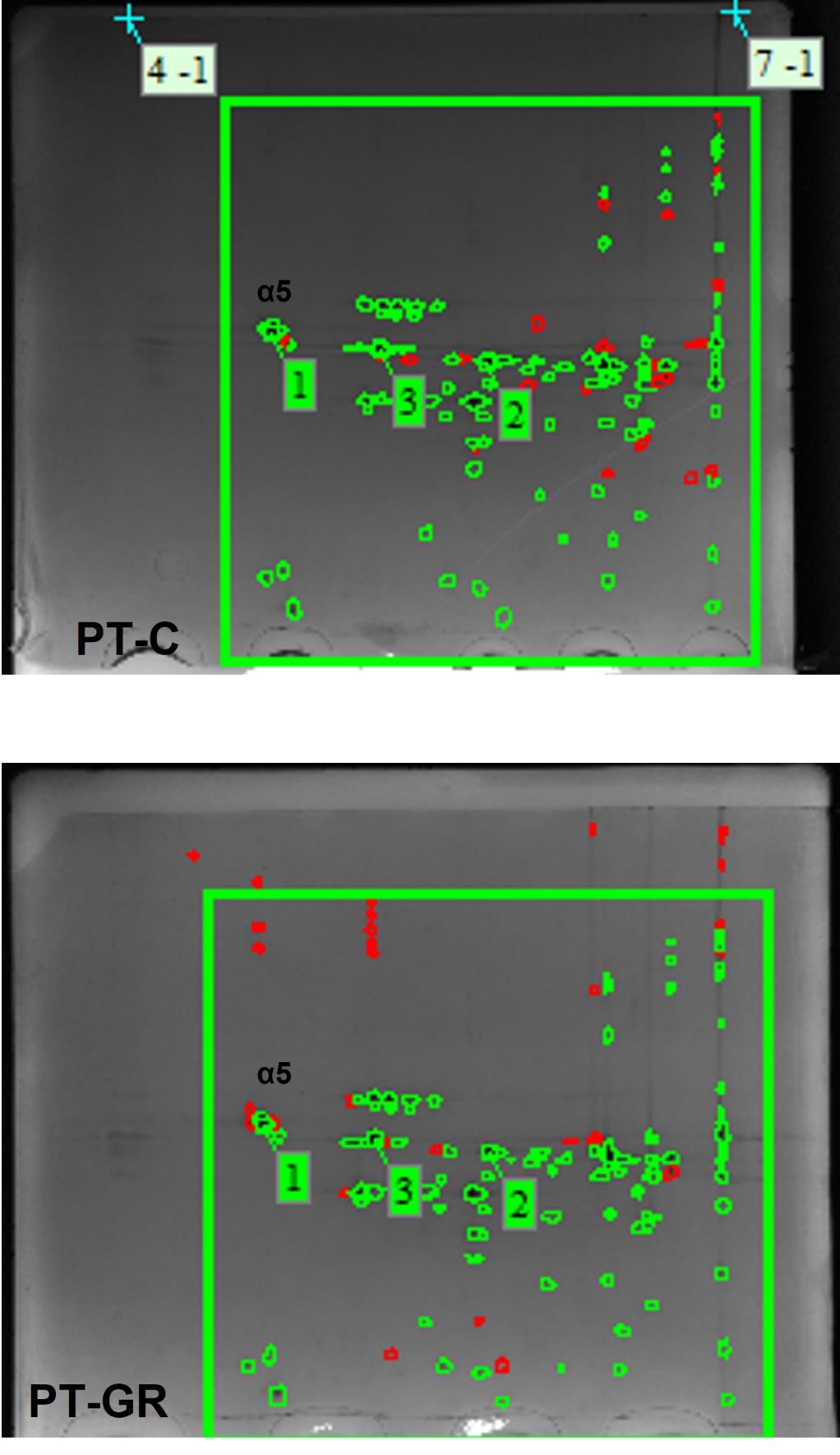
**

Supplementary figure 2. One hundred and fifteen µg of proteasomes isolated from control (PT-C) or glucose restricted (PT-GR) cells were separated according to their isoelectric point with the pH range of 4 to 7 from left to right (first dimension), and size (molecular weight - second dimension). The gels were stained with colloidal Coomassie G-250, scanned in a ChemiDoc MP imaging system (Bio-Rad Lab., California, USA), and the migration pattern between C or GR samples was compared using ImageMaster 2D Platinum software (GE Healthcare, Uppsala, Sweden). Spots colored in green are similar, while spots colored in red were different between the samples.

**
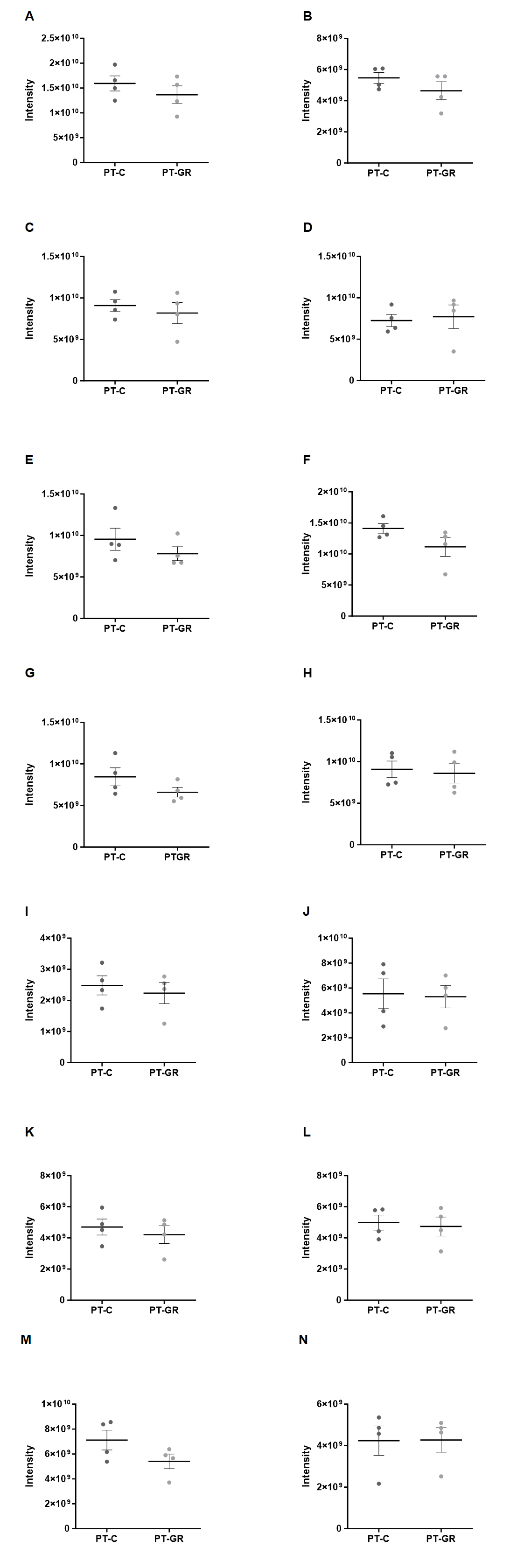
**

**Supplementary Figure 3**

Supplementary figure 3: Glucose restriction does not change the stoichiometry of proteasome subunits. Quantitative analysis of each proteasome subunit: (A-G) alpha subunits in sequence 1 to 7, and (H-N) beta subunits in sequence, 1 to 7. Protein abundances were quantified by the LFQ algorithm, based on the normalized chromatographic peak integrations generated by MaxQuant. Each group represents 4 biological replicates ± standard deviation

**Supplementary Figure 4**

**
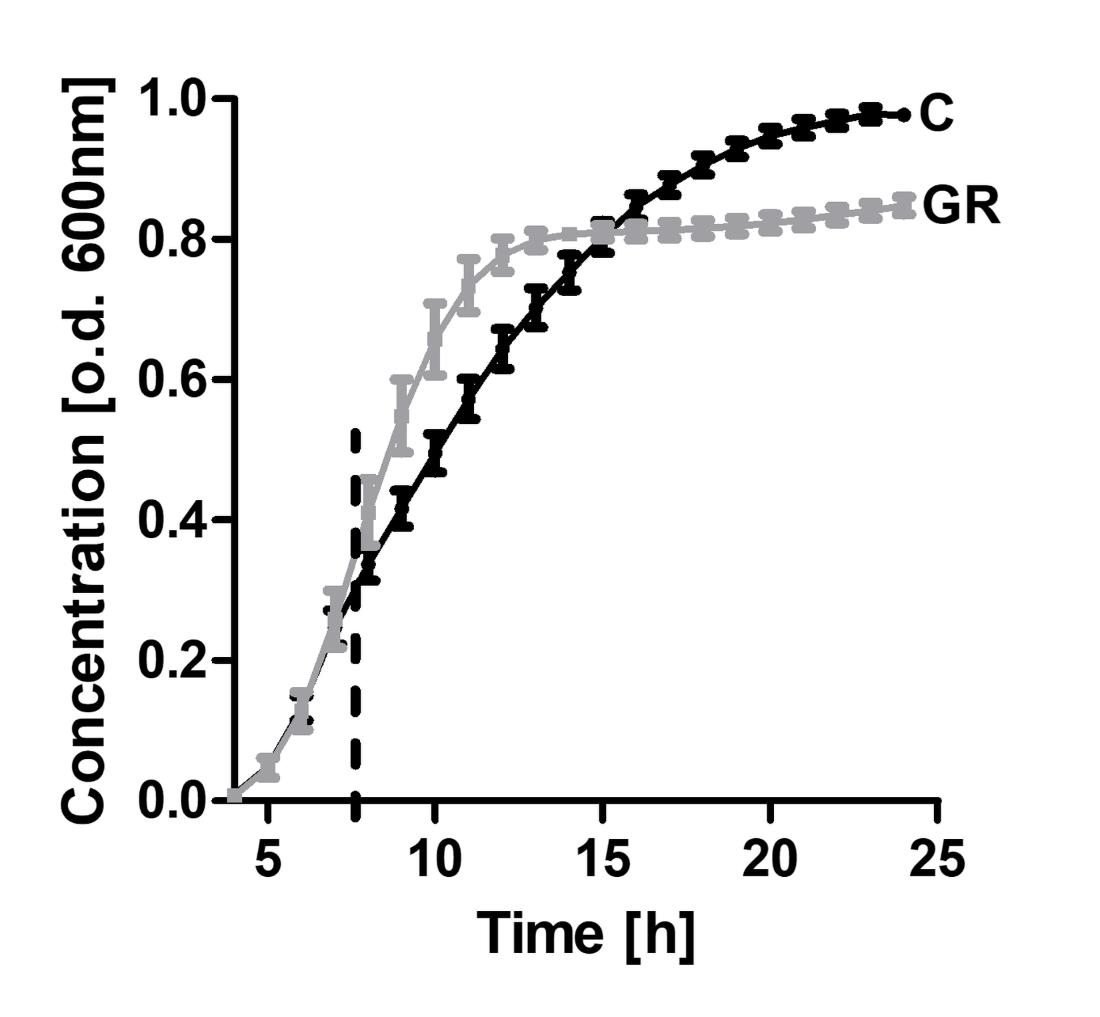
**

Supplementary figure 4: Growth curve of cells grown under control (C) or glucose restriction (GR) condition. Cells were inoculated at the optical density at 600 nm of 0.1. Cell growth was evaluated by measuring the increase of turbidity at 600 nm in an Infinity PRO 200 spectrophotometer (Sunrise®Tecan). The dotted line indicates the time of 7.5 h, chosen for all experiments. Data are represented as the mean ± standard deviation of the mean of an experimental duplicate.

**
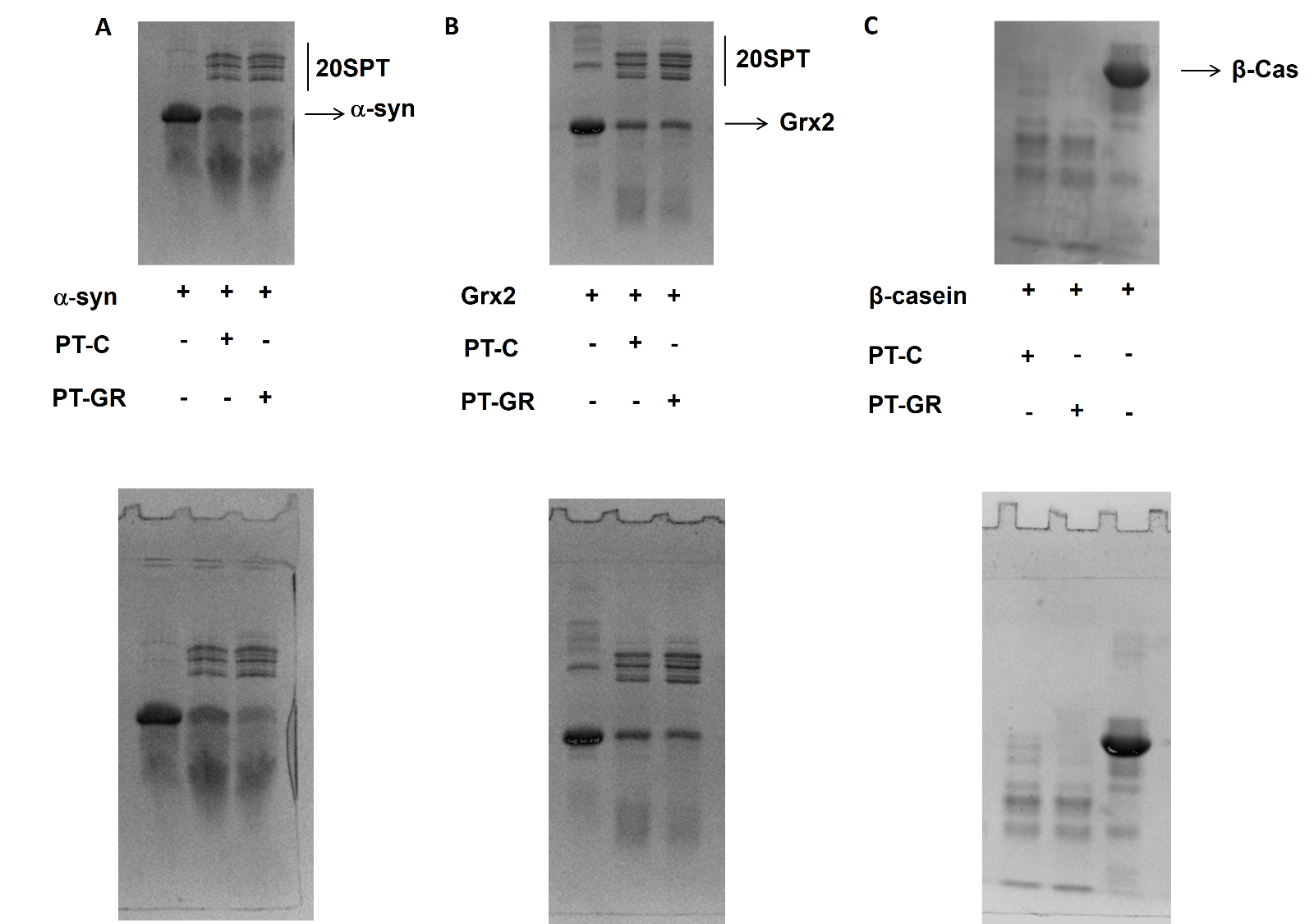
Supplementary Figure 5**

Proteolytic activity of proteasomes isolated from C or GR cells was assessed by incubating 5 µg of proteasomes with α-synuclein (A; single replicate), glutaredoxin 2 (B; single replicate) or β-casein (C; representative gel from 3 biological replicates) as described in Methods. Upper panels are cropped gels, bottom panels, uncropped gels.

**
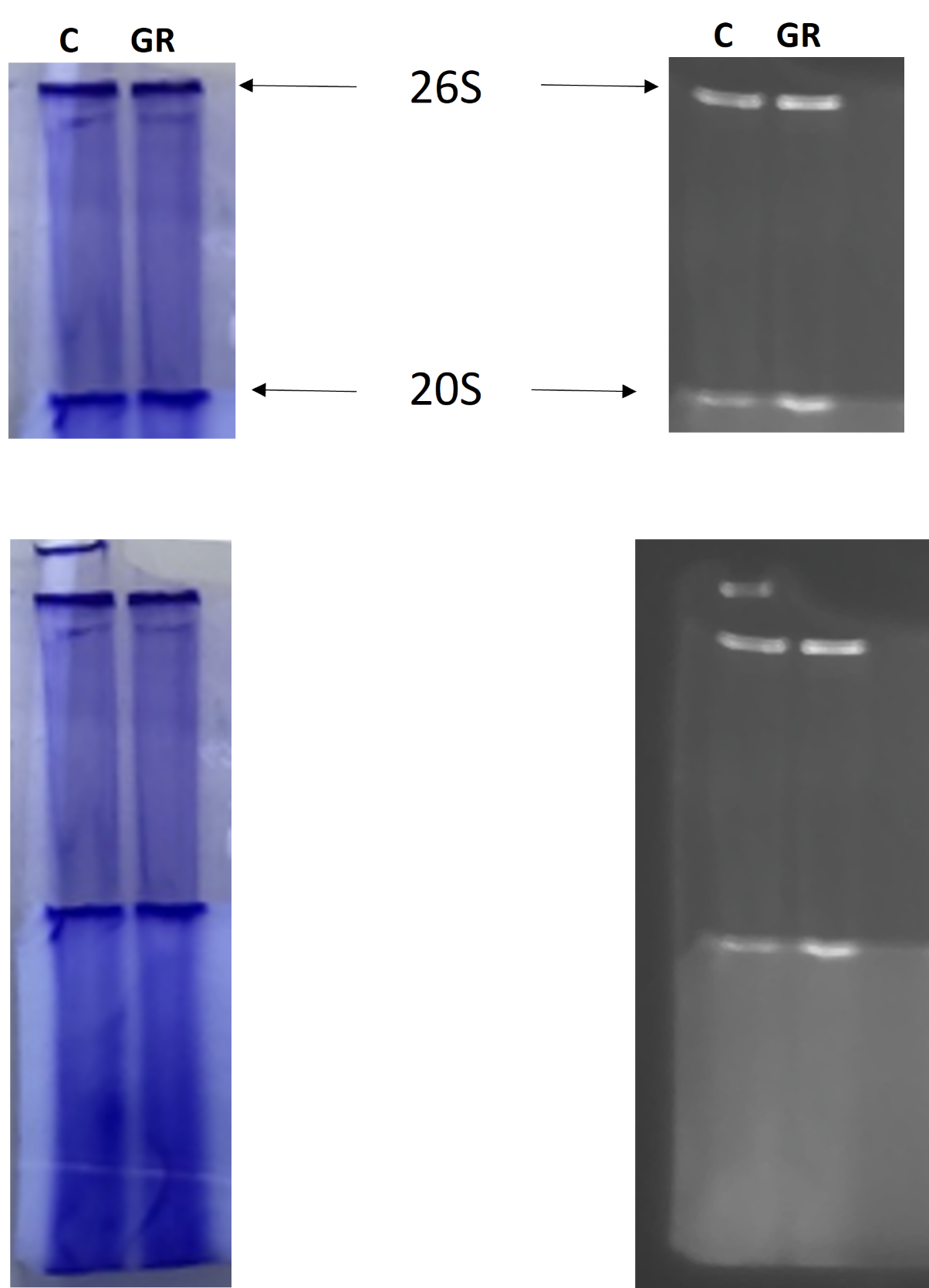
Supplementary Figure 6**

Proteins from cellular extracts obtained from cells grown under C or GR condition were separated by native electrophoresis as described in Methods. The gel was stained with Coomassie (left gels) and in-gel activity was assessed by spreading 100 µM Suc-LLVY-AMC onto the gel (right gels). Upper images are cropped gels as shown in Figure 1, highlighting the 26 and 20 S forms of the proteasome. Bottom images: uncropped gels. Note that in the control sample (C), it is possible to see the 30S form of the proteasome besides the 20S and 26S. We chose not to show this in Figure 1, because all our native gels broke, impairing the imaging of the 30S proteasome in glucose restricted samples.
