# Supplementary material for: Glucose restriction in Saccharomyces cerevisiae modulates the phosphorylation pattern of the 20S proteasome and increases its activity
Source: Sci Rep. 2023 Nov 8;13:19383. doi: 10.1038/s41598-023-46614-x (PMC10632367; doi:10.1038/s41598-023-46614-x)
Supplement: Supplementary file 3 — Supplementary Information 3. [file 41598_2023_46614_MOESM3_ESM.docx]

Supplementary Table 1. Proteins identified by mass spectrometry in control and glucose restricted samples.

| **Gene** | **# of biological replicates in which the protein was found in PT-C samples** | **# of biological replicates in which the protein was found in PT-GR samples** | **Accession # in Uniprot** |
| --- | --- | --- | --- |
| ACT1 | 2 | 3 | P60010 |
| ADE4 | 1 | 1 | P04046 |
| AMD1 | 1 | 1 | P15274 |
| CSF1 | 2 | 1 | Q12150 |
| BLM10 | 4 | 4 | P43583 |
| CAR2 | 1 | 0 | P07991 |
| CDC19 | 1 | 0 | P00549 |
| ENO1 | 1 | 1 | P00924 |
| ERG6 | 1 | 1 | P25087 |
| FKS1 | 1 | 1 | P38631 |
| FUB1 | 4 | 4 | P25659 |
| GPD2 | 2 | 1 | P41911 |
| GSY2 | 1 | 2 | P27472 |
| HSP60 | 3 | 2 | P19882 |
| HSP82 | 1 | 1 | P02829 |
| ILV1 | 1 | 0 | P00927 |
| ILV2 | 1 | 1 | P07342 |
| ILV6 | 1 | 1 | P25605 |
| RPS3 | 1 | 1 | P05750 |
| LYS21 | 1 | 1 | Q12122 |
| PHO84 | 1 | 2 | P25297 |
| PHO88 | 1 | 1 | P38264 |
| PMA1 | 1 | 1 | P05030 |
| PRE1 | 4 | 4 | P22141 |
| PRE10 | 4 | 4 | P21242 |
| PRE2 | 4 | 4 | P30656 |
| PRE3 | 4 | 4 | P38624 |
| PRE4 | 4 | 4 | P30657 |
| PRE5 | 4 | 4 | P40302 |
| PRE6 | 4 | 4 | P40303 |
| PRE7 | 4 | 4 | P23724 |
| PRE8 | 4 | 4 | P23639 |
| PRE9 | 4 | 4 | P23638 |
| PUP1 | 4 | 4 | P25043 |
| PUP2 | 4 | 4 | P32379 |
| PUP3 | 4 | 4 | P25451 |
| RPL12A | 1 | 1 | P0CX53 |
| RPL18A | 1 | 0 | P0CX49 |
| RPL20A | 1 | 2 | P05740 |
| RPL3 | 1 | 2 | P14126 |
| RPL40A | 4 | 4 | P0CH08 |
| RPL4A | 1 | 1 | P10664 |
| RPL9A | 1 | 1 | P05738 |
| RPP0 | 3 | 3 | P05317 |
| RPS0A | 1 | 0 | P32905 |
| RPS18A | 1 | 0 | P0CX55 |
| RPS1A | 1 | 1 | P33442 |
| RPS8A | 1 | 1 | P0CX39 |
| SCL1 | 4 | 4 | P21243 |
| PWP2 | 0 | 1 | P25635 |
| SHM2 | 1 | 0 | P37291 |
| TDH2 | 2 | 3 | P00358 |
| TEF1 | 1 | 2 | P25293 |
| TIF1 | 1 | 2 | P10081 |
| TOM1 | 1 | 2 | Q03280 |
| TY1B-DR4 | 1 | 1 | Q07793 |
| URA2 | 1 | 1 | P07259 |
| VPH1 | 1 | 1 | P43585 |
| YEF3 | 4 | 2 | P32618 |
| YOP1 | 2 | 1 | Q12402 |
| PEX3 | 0 | 1 | P28795 |

Proteasomes were isolated from S. cerevisiae cells cultured under control or glucose restriction, digested with trypsin and analyzed as described in Methods section. Four biological replicates of each experimental condition were analized. Pronteins highlited in grey are 20S proteasome subunits.
