# Supplementary material for: Glucose restriction in Saccharomyces cerevisiae modulates the phosphorylation pattern of the 20S proteasome and increases its activity
Source: Sci Rep. 2023 Nov 8;13:19383. doi: 10.1038/s41598-023-46614-x (PMC10632367; doi:10.1038/s41598-023-46614-x)
Supplement: Supplementary file 6 — Supplementary Information 6. [file 41598_2023_46614_MOESM6_ESM.docx]

Supplementary Table 2

Mass spectrometry data from control (C) or glucose restricted (GR) samples. Proteasomes were isolated from *S. cerevisiae* cells cultured under C or GR, digested with trypsin and analyzed by mass spectrometry as described in Methods. C1, C2, C3 and C4: biological replicates of control samples; GR, GR2. GR3 and GR4: biological replicates of glucose restricted samples.

Supplementary Table 3

Data from phosphopeptides identified by mass spectrometry in control (C) or glucose restricted (GR) samples. Proteasomes were isolated from *S. cerevisiae* cells cultured under C or GR, digested with trypsin and analyzed by mass spectrometry as described in Methods. C1, C2, C3, and C4: biological replicates of control samples; GR, GR2. GR3 and GR4: biological replicates of glucose restricted samples.
